# The Social Determinants of Health in a Cohort of Romanian Patients with Diabetic and Nondiabetic Neuropathy

**DOI:** 10.3390/jcm13226858

**Published:** 2024-11-14

**Authors:** Georgeta Inceu, Adriana Rusu, Norina Alinta Gavan, Cornelia Bala

**Affiliations:** 1 Department of Diabetes and Nutrition Diseases, “Iuliu Hatieganu” University of Medicine and Pharmacy, 2-4 Clinicilor St., 400006 Cluj-Napoca, Romania; inceu.victoria@umfcluj.ro (G.I.); cbala@umfcluj.ro (C.B.); 2 Wörwag Pharma Romania SRL, 21 Garii St., 400267 Cluj-Napoca, Romania; Norina.Gavan@woerwagpharma.ro

**Keywords:** neuropathy, diabetic neuropathy, incidence rate, socioeconomic status, epidemiology

## Abstract

**Background/Objectives**: The importance of the social determinants of health (SODHs) in diabetic and nondiabetic neuropathy has recently gained more attention. This retrospective study examined the correlation of incident diabetic neuropathy and neuropathy of other etiologies with SODH in Romania. **Methods**: All cases with the primary or secondary discharge diagnosis codes of neuropathy reported across Romania in 2019 were analyzed. The crude incidence rate was calculated per 100,000 persons for the whole population resident in Romania on the 1 July 2019. As SODHs sex, gross domestic product (GDP) per capita, unemployment rate, and the number of physicians/1000 persons were evaluated. **Results**: In total, 207,026 hospitalizations with a discharge diagnosis of neuropathy were recorded. Of these, 80,480 had a discharge diagnosis of diabetic neuropathy, with an incidence rate of 414.97 cases/100,000 persons. The incidence rate of diabetic neuropathy by county was correlated with the corresponding GDP (*p* = 0.013) and unemployment rate (*p* = 0.001). By sex, the correlation with GDP remained significant only for women (*p* = 0.010), while the correlation with unemployment rate remained significant in both sexes. No correlation was observed with the number of physicians/1000 persons/county. The incidence rate of neuropathy of other etiology was 652.49 cases/100,000 persons. No correlation between the incidence rate of neuropathy of other etiology by county and the corresponding GDP, unemployment rate or number of physicians/1000 persons was observed neither in the total sample nor by sex. **Conclusions**: Lower socioeconomic status was correlated with a higher incidence rate of hospitalized diabetic neuropathy and not with neuropathies of other etiologies.

## 1. Introduction

Social determinants of health are nonmedical factors recently associated with health outcomes and health disparities [1,2]. According to the Center for Disease Control, social determinants of health are conditions in which people live, learn, work, and age. They shape daily life and include domains such as healthcare quality and access, health literacy, schooling and education quality and access, residential location, and economic stability [1,2]. Social determinants are not limited to the environment, they also include race, gender, and individual socioeconomic status and may explain disparate access to care, health behaviors, and exposure to environmental pollutants, and have a greater influence on health outcomes than genetic and lifestyle factors [1,2].

Despite an increasing body of literature that shows that lower socioeconomic status, unemployment, housing, and food insecurity are associated with poor health outcomes, few studies have tested the association between these social determinants of health and the diagnosis and care of neuropathy (diabetic or other etiologies). The importance of psychosocial and economic factors in diabetic neuropathy has recently gained more attention, and socioeconomic status was found to be a risk factor for diabetic neuropathy in different populations [3,4]. In patients with type 1 diabetes enrolled in the T1D Exchange Clinic Registry from the USA, being a woman and having lower educational attainment and limited access to healthcare, as suggested by the lack of private health insurance, were risk factors for diabetic peripheral neuropathy [3]. In cohorts of Scottish and English patients with type 1 and type 2 diabetes, living in more deprived areas significantly increased the risk of diabetic peripheral neuropathy independent of traditional risk factors such as glycemic control or diabetes duration [4,5].

The relationship with socioeconomic factors is less studied for neuropathies outside diabetes. No data are available on the outcome of alcoholic and uremic neuropathies according to income, neighborhood, or access to healthcare. However, inflammatory neuropathies, such as Guillain–Barré syndrome, have been shown to have a higher frequency in low- and middle-income countries [6]. Similarly, a recent systematic review of 18 studies assessing the UK healthcare system suggests that socioeconomic status is associated with a delay in referral to diagnostic neuroimaging services among communities in lower socioeconomic areas [7].

To date, nothing is known about the association of social determinants of health and neuropathy risk and incidence in Eastern Europe. Understanding how these social determinants influence the incidence of neuropathies could help identify groups at risk of these complications and inform interventions aiming to prevent, diagnose, and treat them. In this context, we here aimed to examine the regional variation in incident diabetic neuropathy and neuropathies of other etiologies and their correlation with social determinants of health in Romania.

## 2. Materials and Methods

To fulfill the objective of this retrospective report we used anonymized data obtained from the National School for Public Health, Management and Health Education (Bucharest, Romania). The National School for Public Health, Management and Health Education is the institution that centralizes all discharges from public hospitals across Romania using the disease related group (DRG) coding system.

All cases reported to this institution between the 1 January 2019 and the 31 December 2019 for hospitalized adult patients identified in the national database using the International Classification of Disease Tenth Revision (ICD-10) primary or secondary discharge diagnosis codes for neuropathy were included in the analysis. Were excluded from the analysis, and thus data were not provided by the National School for Public Health, Management and Health Education, cases in children or cases with a discharge diagnosis code that did not include neuropathy. Diagnosis codes of neuropathy were further classified into diabetic neuropathies (peripheral and autonomic including ICD-10 codes E10.40-E10.43, E10.49, E11.40-E11.43, and E11.49) and neuropathies of other etiologies such as inflammatory, idiopathic, alcoholic, or in other diseases (including ICD-10 codes G60, G61, G62, G62.1, G63, and G64). The full list of ICD-10 codes and the corresponding diseases and health problems is provided in Supplementary Table S1. Only the number of cases for each diagnosis code, age group, legal sex category (named sex hereafter), and county of origin was available, and no patient level data were provided.

The crude incidence rate of hospitalizations with a discharge diagnosis of neuropathy was calculated per 100,000 persons for the whole population resident in Romania on the 1 July 2019 and by sex, age group, and county using the data available on the National Institute of Statistics (Bucharest, Romania) webpage [8].

Starting from the Healthy People conceptual framework [1], we investigated as social determinants of health sex, gross domestic product (GDP) per capita, and unemployment rate as indicators of economic stability, and the number of physicians per 1000 persons as an indicator of healthcare system access and quality. The GDP per capita per county for 2019, the number of physicians, and the number of employed and unemployed persons in each county in the same year were also obtained from the National Institute of Statistics webpage [8]. For GDP per capita, data were available in Romanian Leu and were converted to USD according to the average exchange rate of the National Bank of Romania (Bucharest, Romania) for the year 2019 [9]. The number of physicians per 1000 persons was also calculated for each county. The unemployment rate per county was calculated as the number of unemployed/(number of employed + unemployed) × 100%.

### Statistical Analysis

No power calculation was performed and all cases in adult patients with a discharge diagnosis of neuropathy recorded in all public hospitals in Romania between the 1 January 2019 and the 31 December 2019 and reported to the School for Public Health, Management and Health Education were included in the analysis. Summaries of data were generated using descriptive statistics and the correlation between incident cases of diabetic neuropathy, neuropathy of other etiology, GDP per capita, unemployment rate, and the number of physicians per 1000 persons was tested with Spearman’s ρ. Statistical analysis was performed with IBM SPSS Statistics for Windows, Version 26.0 (Armonk, NY, USA: IBM Corp) and a *p*-value < 0.05 was considered statistically significant.

## 3. Results

**Figure 1:**
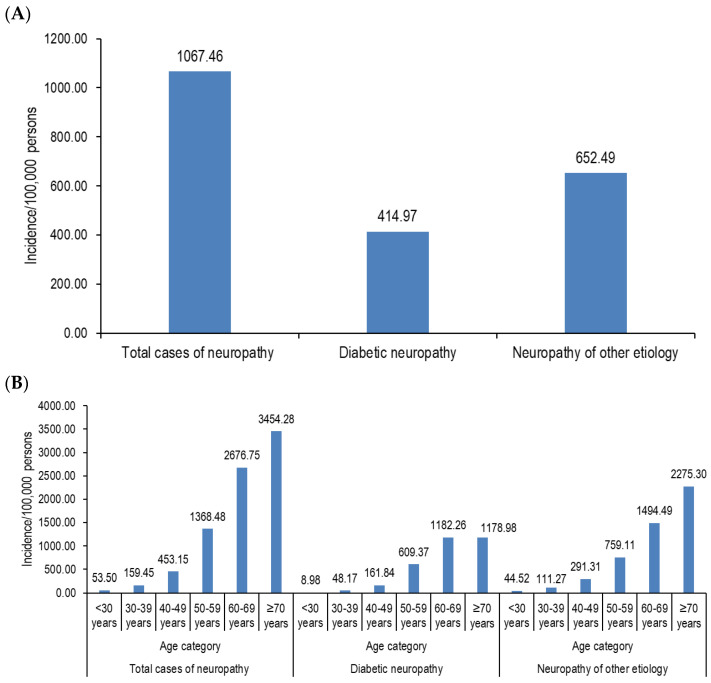

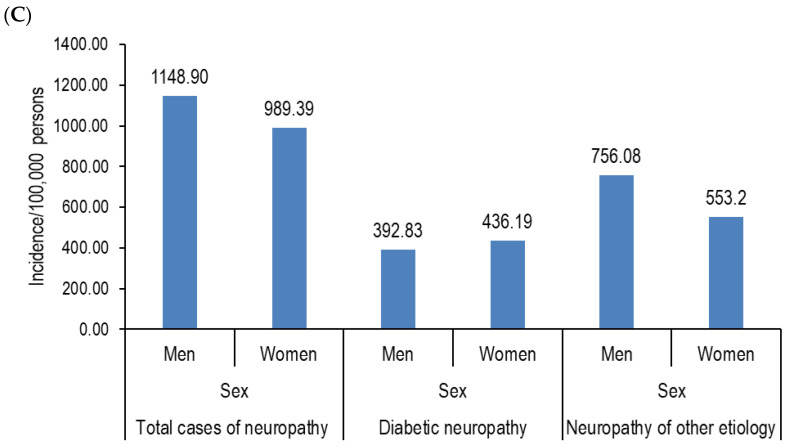
Incidence of hospitalized cases with a discharge diagnosis of neuropathy—total cases (**A**), by age category (**B**), and sex (**C**).

In 2019, a total of 207,026 hospitalizations with a discharge diagnosis of neuropathy were recorded in Romania. The incidence rate per 100,000 persons increased with age from 53.50 in those <30 years of age to 3454.28 in those ≥70 years of age and was higher in men than in women (Figure 1 and Supplementary Table S2).

### 3.1. Diabetic Neuropathy

**Figure 2:**
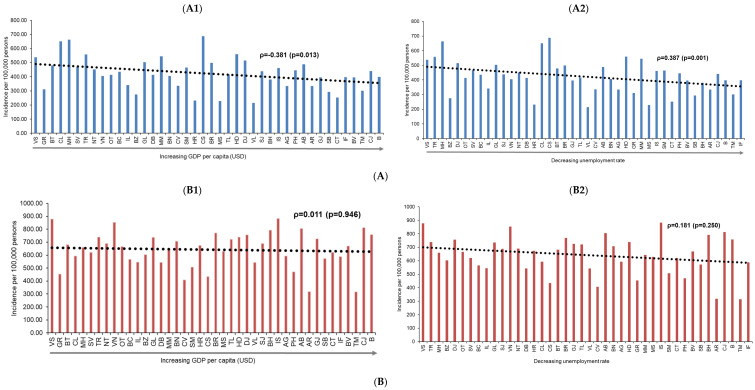
Variation in incident hospitalized cases with a discharge diagnosis of diabetic neuropathy (**A**) and neuropathies of other etiologies (**B**) by county of origin, sorted according to the gross domestic product per capita (**A1**,**B1**) and according to the unemployment rate (**A2**,**B2**). GDP = gross domestic product; USD = US dollar; ρ = Spearman’s ρ correlation coefficient.

Of the total neuropathy cases, 80,480 had a discharge diagnosis of diabetic neuropathy, accounting for an incidence rate of 414.97 cases/100,000 persons. The incidence rate also increased with increasing age, but the incidence rate was higher in women than in men (the men-to-women ratio was 0.86, Supplementary Table S1). The incidence rate of diabetic neuropathy per 100,000 persons varied between counties, from the lowest at 214.37 to the highest at 687.50 (Figure 2A).

An inverse correlation was found between the incidence rate of diabetic neuropathy by county and corresponding GDP (correlation coefficient −0.381, *p* = 0.013). When data were analyzed separately according to the patient’s sex, the correlation between the incidence rate of diabetic neuropathy by county and the corresponding GDP remained significant for women (correlation coefficient −0.393, *p* = 0.010), but not for men (*p* = 0.065). Additionally, a positive correlation between the incidence rate of diabetic neuropathy by county and the corresponding unemployment rate was observed in the total sample (correlation coefficient 0.387, *p* = 0.001), as well as in men and women (*p*-value < 0.05 for both). No correlation was observed between the incidence rate of diabetic neuropathy by county and the corresponding number of physicians per 1000 persons (Table 1).

### 3.2. Neuropathy of Other Etiology

Of the total admitted cases with a discharge diagnosis of neuropathy in 2019, 126,546 were for neuropathies of other etiologies, with an incidence rate of 652.49 cases/100,000 persons. For these cases, the incidence rate also increased with age, while by sex it was higher in men than in women (the men-to-women ratio was 1.31, Supplementary Table S1). A variation in the incidence rate per 100,000 persons among counties was also observed. However, no correlation between the incidence rate of neuropathy of other etiology by county and the corresponding GDP, unemployment rate, or the number of physicians per 1000 persons was observed, either in the total sample or by sex (Figure 2B, Table 1).

## 4. Discussion

The main finding of our analysis was that a lower GDP per capita and a higher unemployment rate were associated with a higher crude incidence rate of hospitalized cases of diabetic neuropathy. No correlation with the incidence rate of hospitalized cases of neuropathies of other etiologies was observed.

Information on the socioeconomic status and the risk of diabetic neuropathy is available but still limited. The analysis of a type 1 diabetes cohort from Scotland showed that living in deprived areas doubled the odds of having diabetic peripheral neuropathy independent of age, diabetes duration, and sex [4]. Similarly, among US adults with type 1 diabetes from the T1D Exchange Clinic Network, a lower educational attainment and lack of private medical insurance, as indicators of socioeconomic status, were associated with higher odds of distal symmetric polyneuropathy [3]. Conflicting reports are available for type 2 diabetes. A UK study showed that treatment for painful neuropathy was more likely in persons living in the most deprived areas [5], while another UK-based study reported no relationship according to patient gender [10]. Several factors have been proposed to explain the association, such as the low availability of healthcare resources, low patient self-efficacy, and higher HbA1c values in the more deprived populations [11].

Data on the relationship between neuropathies of other etiology and socioeconomic status are scarce. We found reports on Guillain–Barré syndrome having a higher frequency in low- and middle-income countries due to poor hygiene, high exposure to infections, and limited access to healthcare facilities [6]. A recent analysis of the UK Biobank data also reported higher deprivation in those with neuropathic pain compared to those without [12]. An explanation was provided by a systematic review of studies analyzing the access to imaging services, which showed a delay in the referral to the neuroimaging services explained by the lack of general practitioner referrals and a delay in health-seeking behaviors among the socially deprived communities in the UK [7]. In our study, we found no correlation between the economic status, as suggested by the GDP per capita, and hospitalized cases of neuropathies of other etiologies. This lack of association should be interpreted together with access to the healthcare system, as indicated by the number of physicians per 1000 persons, for which we also found no correlation. We hypothesize that, due to the limited health literacy regarding neuropathic symptoms, patients may delay/lack health-seeking behaviors for neuropathy of other etiologies.

In our study, data on glycemic control, which is an important risk factor for diabetic neuropathy, were not available [13]. We also lack data on the average age at county level. Age is an important risk factor for diabetic [13] and nondiabetic neuropathies [14]. Counties with aging populations may report a lower GDP per capita due to old age pensions providing a lower income as compared to wage rates and, thus, representing a cofounding factor which should be accounted for. Hypertension, obesity, uremia, smoking, vitamin deficiency, malnutrition, and neoplasms are other risk factors associated with an increased risk of diabetic and nondiabetic neuropathies [14,15], and which were not considered in our analysis. Data from socioeconomically deprived groups and racial and ethnic minorities showed a higher frequency of increased blood pressure, obesity, malnutrition, and diabetes-related chronic complications [16,17,18], explained by a lower socioeconomic status leading to decreased healthcare use, decreased access to care, lower adherence to screening recommendations, and worse health outcomes [19,20,21]. Air pollution is another risk factor for neuropathy [22]. Low-income communities are in general disproportionately exposed to air pollution and environmental toxicants due to inequity in goods and services, nearby pollution sources, and low enforcement of regulations in these communities [23]. We were not able to assess all of these possible confounding factors in our studies due to a lack of or very limited data at the national and county levels. However, we expect that a higher GDP per capita and a lower unemployment rate represent a fair reflection of a good health education and increased healthcare use, screening for diabetes chronic complications, drug access for comorbidities, and a safer environment, and that the lack of the aforementioned data would have a limited impact on our results.

In our study, we used the number of physicians per 1000 persons as an indicator for healthcare system access and we found no correlation between this number and the incidence rate of diabetic neuropathy, suggesting that healthcare access does not explain the increased incidence of hospitalized cases of diabetic neuropathy observed in the more economically deprived counties. Probably other factors that were not assessed, pertaining to the healthcare system (such as quality of care or waiting times) or patients (education, health literacy, health behaviors, and financial reasons that limit outpatient access to medication), may cofound the results and explain the lack of correlation observed.

We also found a higher incidence rate of hospitalized cases of diabetic neuropathy in women and a higher incidence rate of hospitalized cases of neuropathies of other etiologies in men. Female sex has not been associated with a higher prevalence of diabetic neuropathy in previous studies [24,25,26,27]. However, previous studies have shown that a higher proportion of women report painful symptoms of neuropathy despite a higher frequency of diagnosed neuropathy in men [26,28]. A large cohort study enrolling over 15,000 patients with diabetes from England found that women had a 50% higher risk of painful diabetic neuropathy compared to men, independent of age and diabetes duration [26]. Another study showed that women with diabetes report a higher intensity of pain despite a milder form of neuropathy [29]. More recently, data from the EURODIAB prospective diabetes complication study confirmed that female sex is a risk factor for the development of painful diabetic neuropathy in type 1 diabetes, independent of diabetes duration and glycemic control [30]. These painful symptoms often drive the patient to seek medical care and may explain the higher incidence rate of hospitalized cases with diabetic neuropathy in women observed in our sample. Although our study cannot exclude the existence of barriers to accessing quality healthcare in Romania, a considerable body of literature points towards an increased prevalence of chronic pain in women [31]. The mechanisms involved in sex differences observed in chronic pain are not completely understood, and sex hormones are hypothesized to play a key role in pain modulation [30]. Estrogen levels associated with increased pain sensitivity, the sex-dependent effect of microglia and T cells on pain modulation, and cerebral differences in brain regions involved in nociception processing are among the biological mechanisms proposed [32,33,34]. Cultural and psychosocial factors have also been proposed as an explanation for sex differences in pain perception, including gender roles and maladaptive coping strategies [30].

The higher incidence rate of hospitalized cases of neuropathies of other etiologies in men than in women is similar to the one reported in studies that assessed peripheral neuropathy in patients without diabetes [35,36]. Our results may be explained by a higher prevalence in the male population of two major causes of peripheral neuropathy outside diabetes in Romania—alcohol consumption [37] and cancer [38].

We acknowledge certain limitations of our study. First, individual-level data on age, sex, and medical history were not available. Thus, age- and sex-adjusted incidence could not be calculated as the influence of traditional risk factors on the incidence rate could not be assessed. These traditional risk factors might have changed the associations observed between the social determinants of health and hospitalizations. Also, another limitation is the lack of data on educational attainment and income per person at county level. These are social determinants which have been linked to health access disparities in other studies [39] and which might influence the incidence rates of neuropathies in our country. Another limitation is the analysis of hospitalized cases. This underestimates the real incidence of neuropathy in the general population in Romania and future research collecting outpatient data across Romania is warranted to better describe the burden of neuropathy.

## 5. Conclusions

In conclusion, lower socioeconomic status expressed as lower GDP per capita and a higher unemployment rate was correlated with a higher incidence rate of hospitalized diabetic neuropathy. No correlation was observed between economic status and neuropathies of other etiologies. Socioeconomically deprived groups should be targeted for the prevention of diabetic neuropathy and probably all chronic complications of diabetes using available interventions on risk factors to attain recommended objectives. More specifically, increasing social support (family, community, or healthcare-provider support), and the integration of health and social care have the potential to improve diabetes-related outcomes, including clinical and psychosocial ones [23]. Adapting the health messages and health materials to low-literacy communities, increasing awareness of the need for screening for diabetes-related chronic complications and a healthy diet, as well as improved access to quality healthcare and healthier food choices, are practical examples of recommended interventions.

## Figures and Tables

**Table 1:** Correlation of incidence rate of hospitalized cases with a discharge diagnosis of neuropathy with social determinants of health analyzed.

	Total Sample	Men	Women
Diabetic neuropathy			
GDP (USD)	−0.381 (0.013)	−0.287 (0.065)	−0.393 (0.010)
Number of physicians/1000 persons	−0.230 (0.143)	−0.158 (0.319)	−0.241 (0.124)
Unemployment rate (%)	0.387 (0.001)	0.377 (0.014)	0.340 (0.028)
Neuropathy of other etiology			
GDP (USD)	0.011 (0.946)	−0.025 (0.873)	0.039 (0.806)
Number of physicians/1000 persons	0.155 (0.325)	0.107 (0.501)	0.182 (0.249)
Unemployment rate (%)	0.181 (0.250)	0.272 (0.082)	0.063 (0.691)

The data in the table present Spearman’s correlation coefficient (*p*-value). GDP = gross domestic product; USD = US dollar, % = percentage.

## Data Availability

Datasets available on request from the authors.

## Supplementary Materials

The following supporting information can be downloaded at: https://www.mdpi.com/article/10.3390/jcm13226858/s1, Table S1: List of ICD-10 diagnosis codes included in the analysis; Table S2: Incidence of hospitalized cases with a discharge diagnosis of neuropathy.
