# Supplementary material for: The Social Determinants of Health in a Cohort of Romanian Patients with Diabetic and Nondiabetic Neuropathy
Source: J Clin Med. 2024 Nov 14;13(22):6858. doi: 10.3390/jcm13226858 (PMC11594769; doi:10.3390/jcm13226858)
Supplement: Supplementary file 1 [file jcm-13-06858-s001.zip › jcm-3265911-supplementary.pdf]

Supplementary Material

Supplementary Table S1. List of ICD-10 diagnosis codes included in the analysis

ICD-10 code	Disease and medical condition
E10.40	Type 1 diabetes mellitus with diabetic neuropathy, unspecified
E10.41	Type 1 diabetes mellitus with diabetic mononeuropathy
E10.42	Type 1 diabetes mellitus with diabetic polyneuropathy
E10.43	Type 1 diabetes mellitus with diabetic autonomic (poly)neuropathy
E10.49	Type 1 diabetes mellitus with other diabetic neurological complication
E11.40	Type 2 diabetes mellitus with diabetic neuropathy, unspecified
E11.41	Type 2 diabetes mellitus with diabetic mononeuropathy
E11.42	Type 2 diabetes mellitus with diabetic polyneuropathy
E11.43	Type 2 diabetes mellitus with diabetic autonomic (poly)neuropathy
E11.49	Type 2 diabetes mellitus with other diabetic neurological complication
G60	Hereditary and idiopathic neuropathy
G61	Inflammatory polyneuropathy
G62	Other and unspecified polyneuropathies
G62.1	Alcoholic polyneuropathy
G63	Polyneuropathy in diseases classified elsewhere
G64	Other disorders of peripheral nervous system

Supplementary Table S2. Incidence of hospitalized cases with a discharge diagnosis of neuropathy.

	N	Incidence/100,000 persons
**Total cases of neuropathy**	207,026	1,067.46
Age		
<30 years	3,312	53.50
30-39 years	4,316	159.45
40-49 years	13,779	453.15
50-59 years	34,326	1,368.48
60-69 years	67,513	2,676.75
≥70 years	83,780	3,454.28
Sex		
Men	109,056	1,148.90
Women	97,970	989.39
**Diabetic neuropathy**	80,480	414.97
Age		
<30 years	556	8.98
30-39 years	1,304	48.17
40-49 years	4,921	161.84
50-59 years	15,285	609.37
60-69 years	29,819	1,182.26
≥70 years	28,595	1,178.98
Sex		
Men	37,288	392.83
Women	43,192	436.19
**Neuropathy of other etiology**	126,546	652.49
Age (years)		
<30 years	2,756	44.52
30-39 years	3,012	111.27
40-49 years	8,858	291.31
50-59 years	19,041	759.11
60-69 years	37,694	1,494.49
≥70 years	55,185	2,275.30
Sex		
Men	71,768	756.08
Women	54,778	553.20

[1] N=number.
